# Supplementary material for: Application of UPLC-QTOF-MS Based Untargeted Metabolomics in Identification of Metabolites Induced in Pathogen-Infected Rice
Source: Plants (Basel). 2021 Jan 22;10(2):213. doi: 10.3390/plants10020213 (PMC7910874; doi:10.3390/plants10020213)
Supplement: Supplementary file 1 [file plants-10-00213-s001.pdf]

Supplementary Materials

Application of UPLC-QTOF-MS based untargeted metabolomics in identification of metabolites induced in pathogen-infected rice

Mira Oh[a], SeonJu Park[b], Hun Kim[c], Gyung Ja Choi[c], and Seung Hyun Kim[a,*]

[a] *College of pharmacy, Yonsei Institute of Pharmaceutical Sciences, Yonsei University, Incheon, Korea*

[b]*Chuncheon Center, Korea Basic Science Institute (KBSI), Chuncheon 24341, Korea*

[c]*Center for Eco-friendly New Materials, Korea Research Institute of Chemical Technology, Daejeon 34114, Korea*

[*]Corresponding author

Seung Hyun Kim

Tel : +82-32-749-4514

Fax : +82-32-749-4105

E-mail : kimsh11@yonsei.ac.kr

Figure captions

Figure S1. Representative LC-MS chromatographic profiling of control and pathogen-infected rice in negative ion mode. (A,B) Total ion current (TIC) chromatograms of control (A) and infected (B) rice.

Figure S2. Box plot of twelve induced metabolites in infected rice. The bar plots on the left show the original values (mean +/- SD). The box and whisker plots on the right summarize the normalized values. green, control; orange, infected rice.

Figure S1.

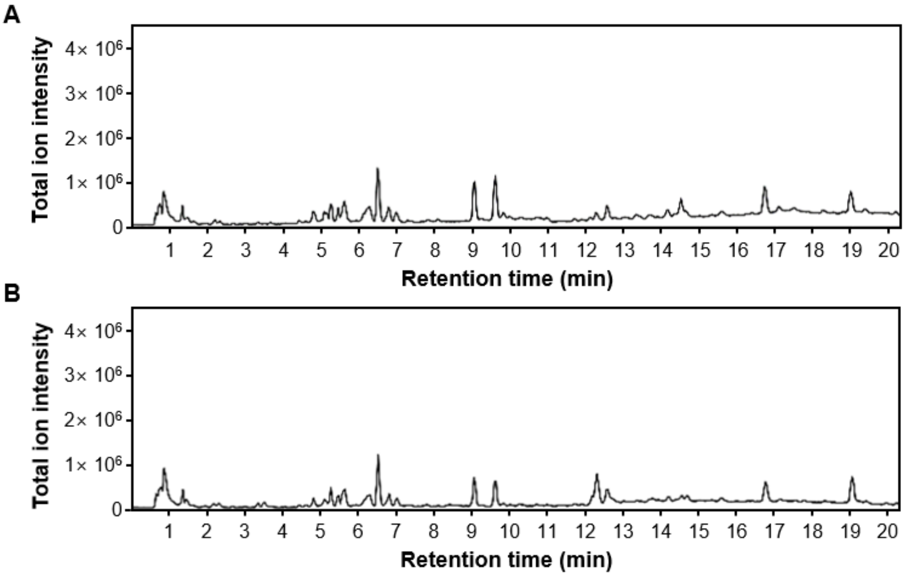
A
Total ion intensity
4× 10⁶
3× 10⁶
2× 10⁶
1× 10⁶
0
1 2 3 4 5 6 7 8 9 10 11 12 13 14 15 16 17 18 19 20
Retention time (min)
B
Total ion intensity
4× 10⁶
3× 10⁶
2× 10⁶
1× 10⁶
0
1 2 3 4 5 6 7 8 9 10 11 12 13 14 15 16 17 18 19 20
Retention time (min)

Figure S2.

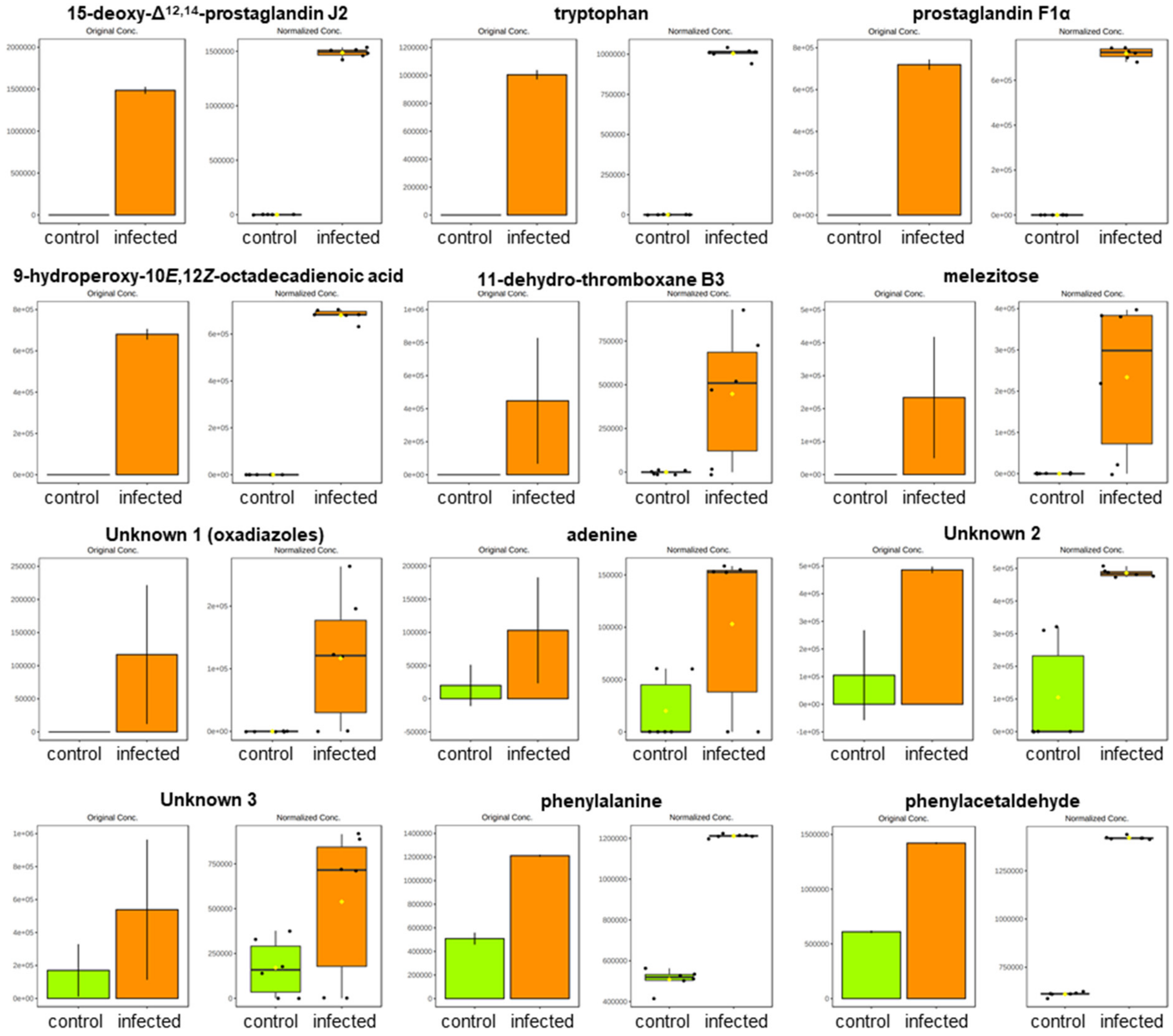
15-deoxy-Δ12,14-prostaglandin J2
tryptophan
prostaglandin F1α
9-hydroperoxy-10E,12Z-octadecadienoic acid
11-dehydro-thromboxane B3
melezitose
Unknown 1 (oxadiazoles)
adenine
Unknown 2
Unknown 3
phenylalanine
phenylacetaldehyde
Original Conc.
Normalized Conc.
control
infected

Table S1. Standardized compound labels in HMDB, KEGG, and PubChem

No	compound ID	HMBD ID	KEGG ID	PubChem CID
1	15-deoxy-$\Delta^{12,14}$-prostaglandin J2	HMDB0005079	C14717	5311211
2	tryptophan	HMDB0000929	C00078	6305
3	prostaglandin F1α	HMDB0002685	C06475	5280939
4	9-hydroperoxy-10*Z*,12*E*-octadecadienoic acid	HMDB0062434	C14827	9548877
5	11-dehydro-thromboxane B3	-	-	16061115
6	melezitose	HMDB0011730	C08243	92817
7	Unknown 1	-	-	-
8	adenine	HMDB0000034	C00147	190
9	Unknown 2	-	-	-
10	Unknown 3	-	-	-
11	phenylalanine	HMDB0000159	C00079	6140
12	phenylacetaldehyde	HMDB0006236	C00601	998
